# Initial Crack Propagation and the Influence Factors of Aircraft Pipe Pressure

**DOI:** 10.3390/ma12193098

**Published:** 2019-09-23

**Authors:** Fusheng Wang, Zheng Wei, Pu Li, Lingjun Yu, Weichao Huang

**Affiliations:** 1 School of Mechanics, Civil Engineering and Architecture, Northwestern Polytechnical University, Xi’an 710129, China; wz199477@mail.nwpu.edu.cn (Z.W.); wchuang@mail.nwpu.edu.cn (W.H.); 2 School of Aeronautics, Northwestern Polytechnical University, Xi’an 710129, China; zhongzhoulipu@mail.nwpu.edu.cn; 3 Department of Aeronautical Machinery Engineering of Army Aviation Institute, Beijing 101123, China; yu_lj10@sina.com

**Keywords:** internal pressure, crack propagation, the extended finite element method, initial crack, influence factors

## Abstract

In aircraft engineering, an increase of internal pressure in a hydraulic pipe increases the probability of pipe damage, leading to crack propagation becoming a serious issue. In this study, the extended finite element method (XFEM) is applied to simulate initial crack propagation in hydraulic pipes and to investigate the influence factors. Stress intensity factors are extracted to verify the mesh independence of XFEM, which is based on the level set method and unit decomposition method. A total of 30 finite element models of hydraulic pipes with cracks are established. The distribution of von Mises stress under different initial crack lengths and internal pressures is obtained to analyze the change of load-carrying capacity in different conditions. Then, a total of 300 finite element models of hydraulic pipes with different initial crack sizes and locations are simulated under different working conditions. The relationship between the maximum opening displacement and crack length is analyzed by extracting the opening displacement under different initial crack lengths. The length and depth of the initial crack are changed to analyze the factors affecting crack propagation. The opening size and crack propagation length are obtained in different directions. The results show that radial propagation is more destructive than longitudinal propagation for hydraulic pipes in the initial stage of crack propagation.

## 1. Introduction

There are many industrial structures that are normally regarded as shells, such as reactor pressure vessels, natural gas pipelines, and aircraft hydraulic pipes. These structural materials containing inclusions or initial cracks may decrease load-carrying capacity in a short time [1]. A substantial decrease in load-carrying capacity will lead to serious malfunction. For example, a series of accidents on the de Havilland Comet airplane happened in the 1950s, in which a main reason for these accidents was crack propagation in hydraulic pipes. In general, it is difficult to ensure that aircraft structures do not engender flaws over their service life. Therefore, the research on structures that contain initial cracks has become very important. Crack propagation path and length in aircraft hydraulic pipes should be further researched in scenarios where load-carrying capacity has decreased, which will guide researchers and engineering technicians to understand the malfunction mechanisms of aircraft hydraulic pipes.

A number of studies on initial crack propagation in actual structures have been done. Experimental methods can display the crack propagation procedure and results directly and accurately. For example, an experimental study on fatigue life and crack propagation in bolted joints under tightened torque indicated that the component lifespan under low amplitude load is longer than that under high amplitude load [2]. Another experiment examined the crack depth ratio threshold in slow crack propagation in high density polyethylene (HDPE) pipes. The results indicated that the threshold is independent of strain rate, crack number, and load types [3]. There are also some researchers who have studied crack propagation in plates based on corrosion fatigue theory. The results showed that cracking is more intense in cases of bending than in cases of tension [4]. On the other hand, numerical methods have become more popular in simulating crack propagation. For example, a numerical analysis based on the detailed finite method was used to analyze notched API X65 pipes with a local fracture criterion model and stress-modified fracture strain model. The results indicated that pipe failure had taken place when the maximum load was attained in the flawed area before global instability. A new method was also proposed to simulate ductile failure using the finite element method (FEM), and simulation results agreed well with experimental data [5,6]. By using the S-Version FEM (S-FEM) technique to model crack propagation under complex loads, such as thermal and residual stress fields, one only needs to re-mesh the local mesh with an auto-meshing technique, after which the crack path can be modeled easily [7]. In order to evaluate fracture parameters more accurately, an interaction integral method is proposed for quadratic tetrahedral finite elements (FE). This method decreases the error of the interaction integral by adding correction terms and by building a new auxiliary solution [8]. Although the finite element method has been widely used to simulate crack propagation, some questions still exist regarding simulation precision. For example, the crack path must coincide with the edges of elements, which limits the degree of freedom in crack propagation. Another limitation of the finite element method is that it is difficult to accurately capture the stress field close to the crack tip because singular points may occur at the crack tip. A solution to deal with this problem is to keep re-meshing to match crack propagation. However, lots of time and computing resources would be occupied.

In order to solve the above problems, a minimal re-meshing finite element method for crack growth was proposed by Belytschko and Black [9]. With this method, the crack is allowed to be arbitrarily aligned within the mesh. However, the re-meshing of the crack tip is difficult. Partition of unity enrichment techniques developed for interface cracks was proposed by Sukumar N et al. [10]. Good agreement between numerical results and reference solutions was achieved for interfacial crack problems. Then, the extended finite element method (XFEM) was proposed based on conventional finite elements, in which a discontinuous enhancement function was added to describe the discontinuous displacement in finite elements, and the unit decomposition method was introduced to describe the crack location [11,12,13,14]. XFEM has a great advantage in simulating crack propagation and does not need to keep re-meshing. At present, this method has been widely used to simulate crack propagation in an arbitrary path, in which a valid idea to update the value of the level set method and a developed penalty function is proposed. This method can also be used to simulate crack propagation in the three-dimensional (3D) domain [15]. Simulation results show that stress intensity factors for both static and growing cracks can be extracted by this method. Zhang et al. [16] studied crack propagation with specified location and initial length through XFEM. The results indicated that a circular embedded crack is more secure than an elliptical embedded crack. Some researchers have studied cracks perpendicular to cross-section or axis, along with the relationship between load and crack propagation path [17,18,19]. Results have shown that damage initiation and resistance ability against crack propagation are significant factors of XFEM that greatly affect the crack behavior and load-carrying capacity of hydraulic pipes. Through studying the angle of the initial crack, the results show that the fatigue life will be shorter when the angle of the initial crack is larger [20]. There are also some researchers who have studyied the effect of residual stress fields on the crack propagation threshold [21]. Results showed that the effect of residual stress on crack propagation is determined by the associated stress intensity factor. Na, Spatari, and Hsuan combined the essential work of fracture (EWF) concept and XFEM to study fracture characterization of HDPE, the results of which showed that extreme failure stress decreases and stress yield increases with the same recycled content [22]. An inclined crack under axial tensile load was studied and validated using XFEM, with numerical results closely agreeing with experimental results [23]. Geniaut et al. [24] described various kinds of new methodologies. A new method named the projection method was proposed to update the level set method, which was re-computed using the true crack opening displacement (COD). Bayesteh and Mohammadi [25] presented a new enrichment function for crack tips to reduce element numbers and raise the parameter accuracy of the computed fracture mechanics. A new method was proposed by Lin et al. to evaluate the goal-oriented error [26]. Results showed that the error range of XFEM simulations can be solved using this method. In addition, a new XFEM method named multi-scale XFEM was proposed, offering great promise in modeling the effects of microstructural features, especially micro-cracks. Multi-scale XFEM emphasizes transformation of variables in multi-scales and the effect of micro-fields in coarse-scale formulation [27]. A method combining the classical global–local finite element method and the partition of unity approach was presented to solve fracture mechanics problems with multiple cracks in the domain [28]. A two-scale, global–local XFEM approach was presented by Malekan et al. [29] to model crack propagation in planes. Crack propagation direction can be determined by the stress intensity factor under linear elastic fracture mechanics, the robustness and accuracy of which are verified by solving several linear elastic fracture mechanics problems. In this paper, propagations of cracks in macrostructures are mainly considered. Therefore, XFEM will be used to simulate crack propagation in aircraft hydraulic pipes under different conditions in this paper. The level set method and unit decomposition will be used to trace and calculate discontinuous displacement. Although many researchers have studied crack propagation in flawed structures through experimental or numerical simulation, studying crack propagation in both longitudinal and radial directions is insufficient. Here, the relationship between longitudinal or radial crack propagation length and initial crack length or depth will be researched. In addition, factors affecting crack propagation along the longitudinal and radial directions will be discussed further.

## 2. The Extended Finite Element Method

The advantages of the XFEM are obvious compared with the conventional finite element method in solving the crack propagation problem. The mesh created by XFEM is independent of geometric or physical interfaces inside the structure, which makes it difficult to perform high-density meshing at high stress and deformation concentrations in the crack tip. The XFEM also makes it possible to simulate crack propagation without re-meshing [9,11]. In this paper, the basic theory of XFEM will be elaborated. Then, method correctness and mesh independence will be verified in Section 2.

### 2.1. Level Set Method and Unit Decomposition Method

The main methods contributing to the XFEM are the level set method and unit decomposition method. The level set method is used to trace discontinuous displacement. It can calculate discontinuous displacement within an unchanged mesh and also can be extended to a multidimensional situation. Discontinuous displacement is described with the function f(x(t),t), which is related to time and space. In discontinuous displacement, all discontinuous points are satisfied with

(1) f(x(t),t)=0

**Figure 1:**
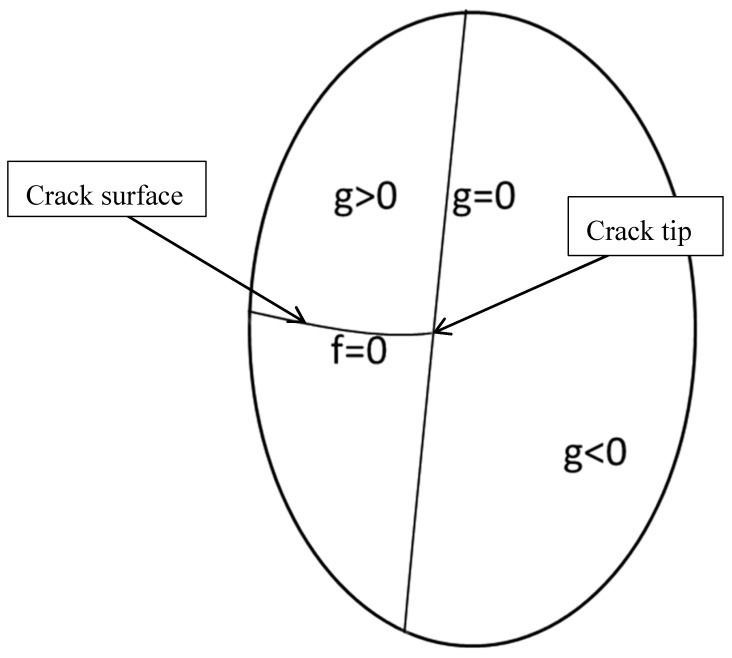
Crack surface described by the level set method.

The level set functions on both sides of the crack have opposite signs. A crack surface is described in Figure 1 [30]. The level set function [24] *g* is used to characterize the position of the crack tip. The crack tip is the point that function *g* is equal to 0.

In order to overcome the difficulty of calculating the discontinuous displacement, the unit decomposition method is imported. Displacement filed in the XFEM can be expressed as

(2) $$U=\sum_{I}N_{I}(x)u_{I}+\psi (x)$$

where $N_{I}$ is the shape function of classical FEM, $u_{I}$ is the nodal freedom degree, and ψ(x) is the enhancement function to improve the discontinuous displacement field. Here, ψ(x) can be expressed as

(3) $$\psi (x)=\sum_{J}N_{J}(x)q_{J}\Phi (x)$$

Then, Equation (2) can be expressed as

(4) $$U=\sum_{I}N_{I}(x)u_{I}+\sum_{J}N_{J}(x)q_{J}\Phi (x)$$

where $q_{J}$ represents the added degree of freedom of the point that does not contain an actual physical meaning. It is used to adjust the enrichment functions Φ(x) to describe displacement that is filed more accurately.

A cracked structure can be summarized as a discontinuous problem, which can be divided into two different types. One type is where an element is pierced by a crack, for which the enhancement shape function can be expressed as

(5) $$\psi_{J}(x)=N_{J}(x)H(f(x))$$

where H(x) is Heaviside function.

(6) $$H(x)=\{\begin{array}{c} 1,\\ -1, \end{array}\begin{array}{cc} & x\geq 0\\ & x<0 \end{array}$$

If the point position is same as the unit normal vector, *x* is positive. Otherwise, it is negative. The other type is where the crack tip impact in the element, for which the enhancement shape function can be expressed as

(7) $$\psi_{J}(x)=N_{J}(x)\Phi (x)$$

Φ(x) is a linear combination of functions, which can be expressed as

(8) $$\Phi (x)=\left[\sqrt{r}\sin\frac{\theta }{2},\sqrt{r}\sin\frac{\theta }{2}\sin\theta ,\sqrt{r}\cos\frac{\theta }{2},\sqrt{r}\cos\frac{\theta }{2}\sin\theta \right]$$

where r and θ are polar coordinate axes at the crack tip.

### 2.2. Correctness and Mesh Independence Verification of the XFEM 

**Figure 2:**
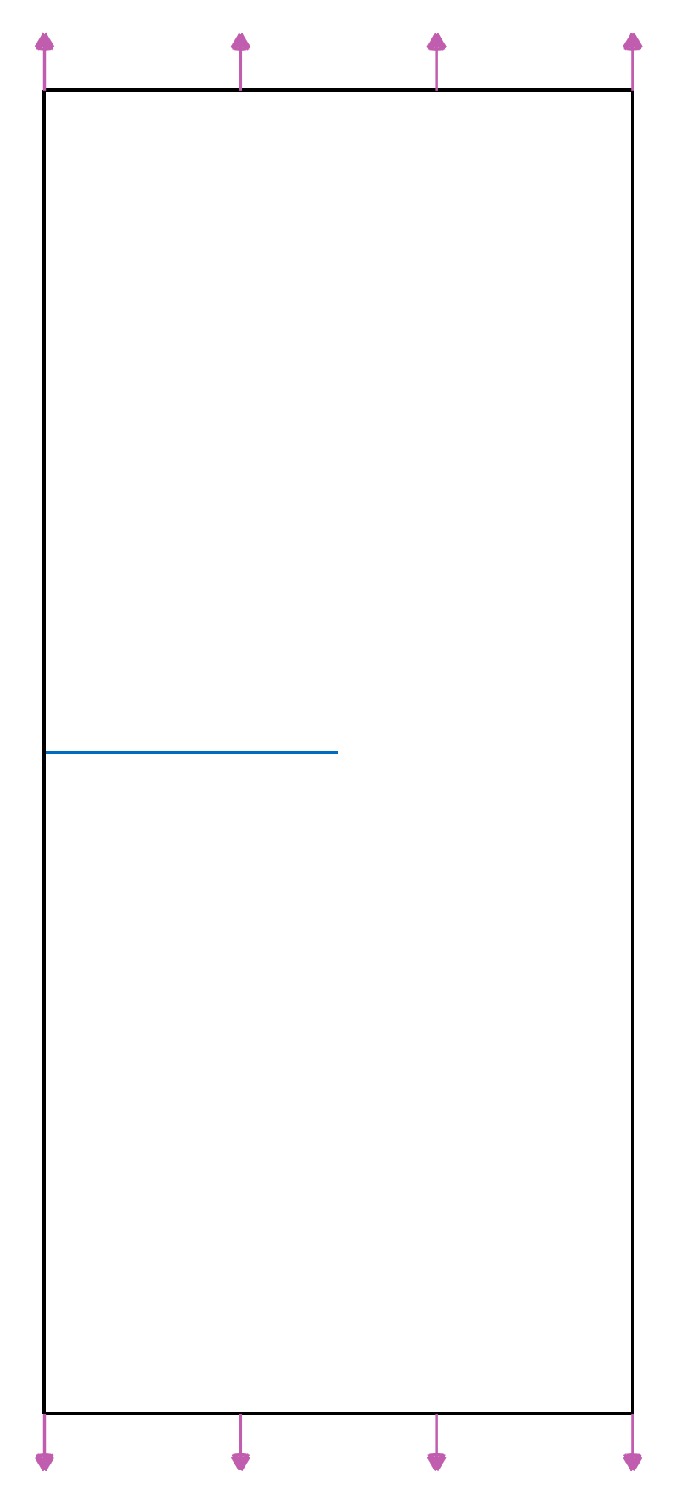
Rectangular plate with a horizontal crack.

The correctness of XFEM should be verified in the 3D domain. A 3D plate that containing a crack in the middle of an edge is shown in Figure 2. The rectangular plate geometry is 18 m × 8 m and the crack lengths vary from 0.5 m to 4 m, with 0.5 m tolerance. The rectangular plate thickness is 1 m and load P, applied to both the top and bottom, is 1 MPa. Load P is applied in the XY plane and restricted in the Z coordinate axis. The material properties of the rectangular plate are given in Table 1. 

**Figure 3:**
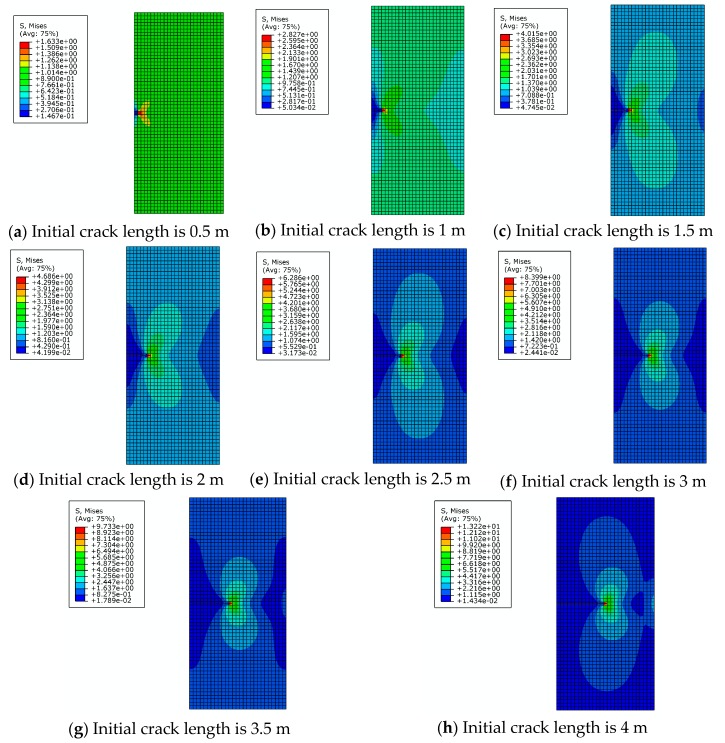
The von Mises stress distribution for different initial crack lengths.

It can be seen from Figure 3 that there is significant stress concentration at the crack tip. The von Mises stress distribution of the entire rectangular plate is axisymmetric and the stress magnitude is inversely proportional to the distance from the crack tip. The value of the von Mises stress at the crack tip increases with increasing crack length when the external force is constant. At both ends of the rectangular plate, the stress magnitude tends to become smaller overall. This shows that the increase of the crack length makes the load-carrying capacity of rectangular plate continue to decrease. When the crack length changes from 0.5 m to 3 m, the position near the intersection of the crack extension line and the rectangular plate is the site where the stress magnitude is smaller. When the crack length increases from 3.5 m to 4 m, the crack opening displacement is increased and the intersection vicinity is squeezed. This results in the stress value exceeding the vicinity area.

Stress intensity factor *F* under this condition is satisfied with

(9) $$K_{I}=F\sigma \sqrt{\pi a}$$

When rotation of the rectangular plate end is not restricted, and when $\frac{h}{b}\geq 1$ and $\frac{a}{b}\leq 0.6$ are satisfied, the stress intensity factor *F* can be expressed as [31]

(10) $$F=1.12-0.23\frac{a}{b}+10.6{\left(\frac{a}{b}\right)}^{2}-21.7{\left(\frac{a}{b}\right)}^{3}+30.4{\left(\frac{a}{b}\right)}^{4}$$

where *h* is the length of the rectangular plate, *b* is the width of the rectangular plate, and *a* is the initial crack length. *F* values calculated by Equation (10) are given in Table 2.

**Figure 4:**
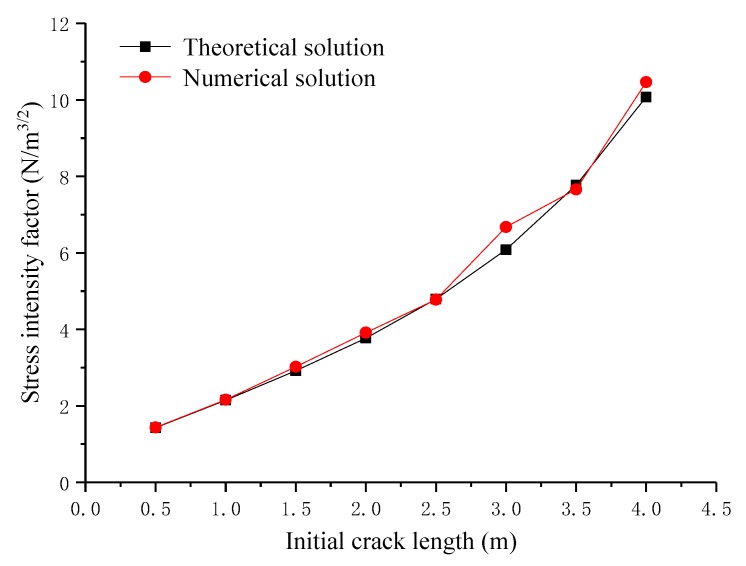
Stress intensity factors for different initial crack lengths.

The effect of the initial crack length on the magnitude of stress intensity factors is shown in Figure 4. Here, *K_I_* is one of the basic parameters in fracture mechanics. The stress field in the crack tip and the J-integral can be derived from *K_I_.* At the same time, the results of numerical solution *K_I_* and theoretical solution *K_I_* agree with each other very well. As shown in Figure 3, distribution of von Mises stress (MPa) is symmetrical and the stress reaches the peak value. It can be seen that simulation results agree with theoretical results, so XFEM has good ability to simulate the 3D crack structure.

**Figure 5:**
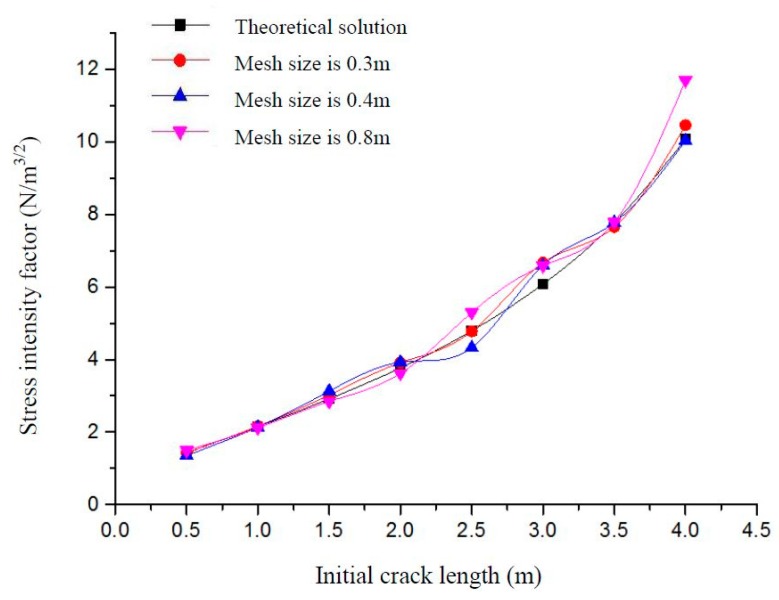
Stress intensity factors for different initial crack lengths and mesh densities.

Three models with different mesh densities are used to verify the mesh independence characteristics of XFEM, which provides the basis for the meshing of finite element models. This is illustrated using the example of a rectangular plate with horizontal cracks in Section 2.2, with mesh sizes mainly selected as 0.3 m, 0.4 m and 0.8 m. The mesh independence of XFEM will be verified by comparing the results of numerical simulation with different mesh sizes. Stress intensity factors under different initial crack lengths and mesh densities are shown in Figure 5. It can be seen that when the mesh density changes from 0.3 m to 0.4 m and 0.8 m, the stress intensity factors of these three cases are close to the theoretical solution. The von Mises stress distribution for different initial crack lengths and mesh sizes is shown in Table 3.

Twenty-four models will be created with three different mesh sizes. Due to the large number of models, only the stress distributions of rectangular plates with crack lengths of 0.5 m, 2 m, and 4 m are shown here. It can be seen that mesh size has little effect on the shape of the stress distribution, especially on the stress concentration area. 

## 3. Crack Propagation in Aircraft Pipes

### 3.1. Geometric and Numerical Models

**Figure 6:**
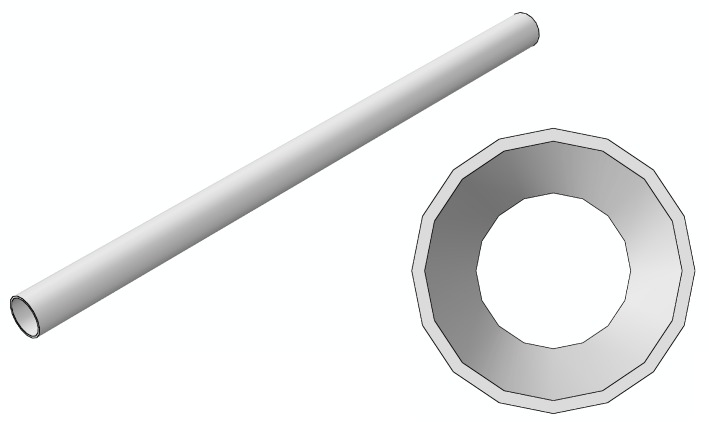
Geometry model of a hydraulic pipe.

**Figure 7:**
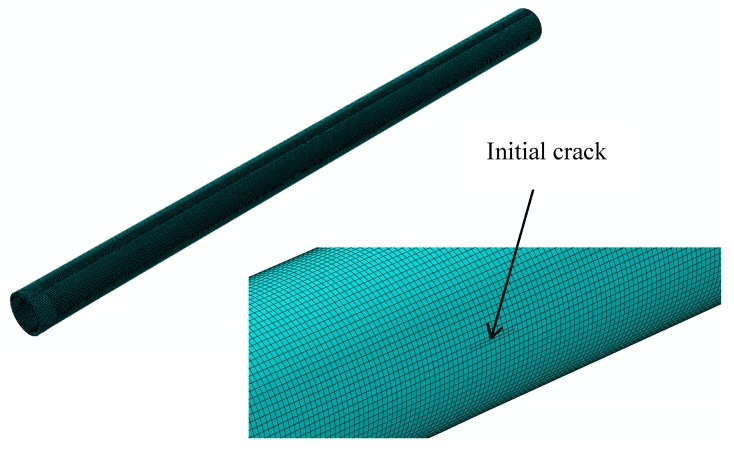
Finite element model of a hydraulic pipe and an enlargement of the initial crack section.

As shown in Figure 6, the hydraulic aircraft pipe simulated in this section is a straight pipe with a length of 300 mm. The crack is longitudinal and propagates through the pipe. The initial crack lengths vary from 1 mm to 10 mm, with intervals of 1 mm. The finite element model of the hydraulic pipe with an initial crack is shown in Figure 7, where the initial crack length is 5 mm. The geometrical dimensions of the model only change in terms of crack length; the other geometric dimensions remain the same. Therefore, only a cracked pipe and its enlarged view are shown in Figure 7—the other nine finite element models are not given. The boundary conditions are fixed constraints. 

Stress—strain fields of a hydraulic pipe with different initial crack lengths under internal pressures of 21 MPa, 28 MPa, and 35 MPa are analyzed and a total of 30 models are calculated. The radius of the pipe model is 8 mm, the wall thickness is 0.8 mm, and the length is 300 mm. The specific mechanical parameters are given in Table 4. The mesh is refined near the initial crack and ten layers are divided in the thickness direction. The element type is solid element C3D8R and total of 104,000 elements are created.

### 3.2. Results and Discussion

**Figure 8:**
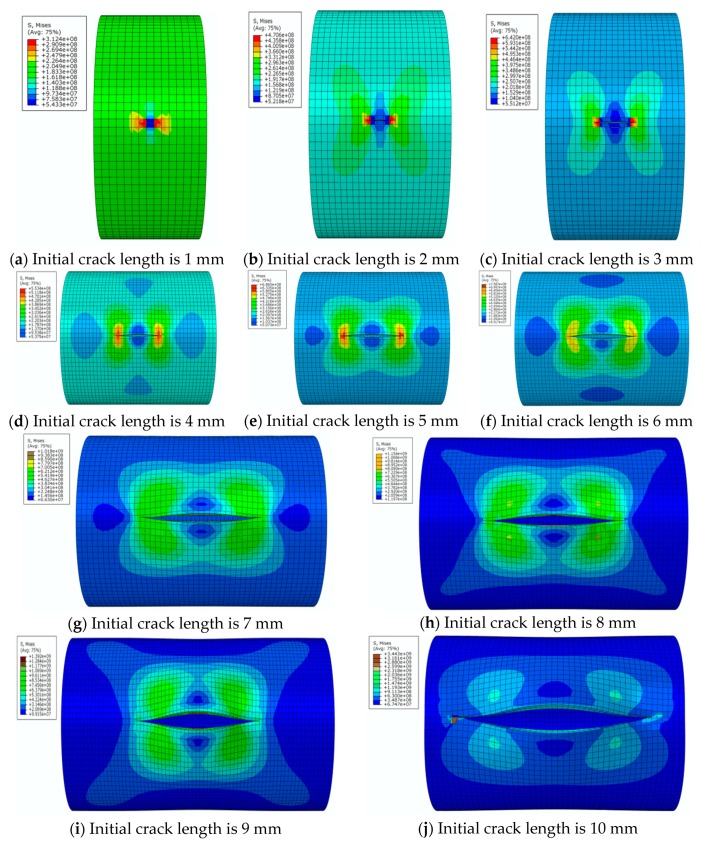
The von Mises stress distribution for different initial crack lengths, where the internal pressure is 21 MPa (here, images are magnified 10 times for visual display).

By analyzing Figure 8, it can be seen that distribution of von Mises stress in the pipe wall changes continuously with the change of crack length under the specified internal pressure. When the crack length changes from 1 mm to 6 mm, the distribution of the high-stress region expands continuously and remains symmetrical. At the same time, two low-stress regions appear along the extension of the crack length. When the crack length changes from 7 mm to 10 mm, both the high-stress and low-stress regions expands rapidly. The low-stress region continuously erodes to the vicinity of the crack tip and the stress value in the low-stress area also decreases. This indicates that the effects of structural rigidity and strength (which are affected by crack length) on the stress distribution are greater than the effect of stress concentration at the crack tip. When the crack length is 10 mm, it is basically considered that the pipe has lost its load-carrying capacity.

When the internal pressure changes from 21 MPa to 28 MPa, and then to 35 MPa, the stress distribution area changes very little, except that the numerical value is different when is crack length is small. The von Mises stress distribution values under pressures of 28 MPa and 35 MPa together with changes of crack length are listed in order, to ensure brevity. Only some cases with special distribution patterns are discussed in detail. 

**Figure 9:**
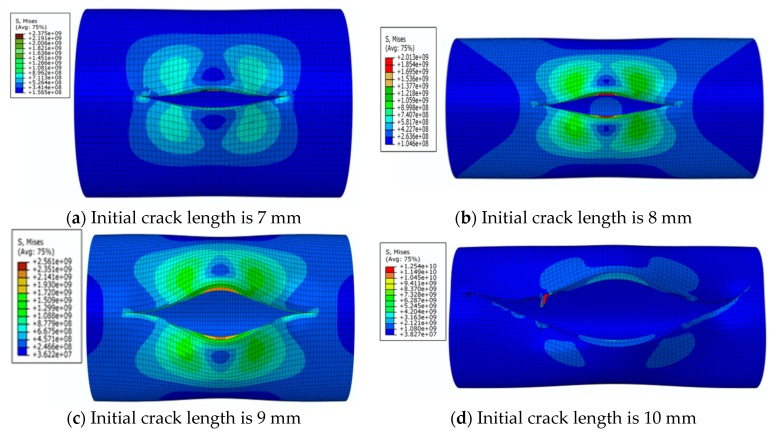
The von Mises stress distribution for different initial crack lengths, where the internal pressure is 28 MPa (here, images are magnified 10 times for visual display).

The von Mises stress distribution is shown in Figure 9a, where initial crack length is 7 mm under an internal pressure of 28 MPa. It can be seen that with the increase of initial crack length and the increase of strain due to crack propagation, the central symmetry of the high-pressure region is destroyed and the crack begins to tilt. The von Mises stress distribution is shown in Figure 9b,c, where the initial crack lengths are 8 mm and 9 mm, respectively. It can be seen that the crack appears to be changing, and the pipe is further destroyed. The crack propagation is shown in Figure 9d, where the initial crack length is 10 mm. It can be seen that pipe completely loses load-carrying capacity due to the crack exceeding the critical value.

**Figure 10:**
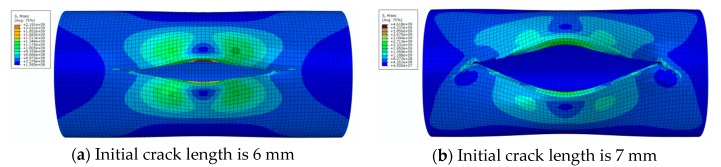
The von Mises stress distribution under different initial crack lengths, where the internal pressure is 35 MPa (here, images are magnified 10 times for visual display).

The von Mises stress distribution under an internal pressure of 35 MPa and with different initial crack lengths is shown in Figure 10. It can be seen that the initial crack appears as a serrated shape when the initial crack length increases to 6 mm during the propagation process, as shown in Figure 10a. The crack extends to the final stage when the initial crack length is 7 mm, as shown in Figure 10b. The serrated shape then becomes more obvious and shows a large tilt angle. Calculation results show that pipe cracks enter a rapid and arbitrary stage of development, and the pipe is completely destroyed when the initial crack length is more than 8 mm. However, pipes in this state have no practical significance in engineering, so these situations are not discussed.

**Figure 11:**
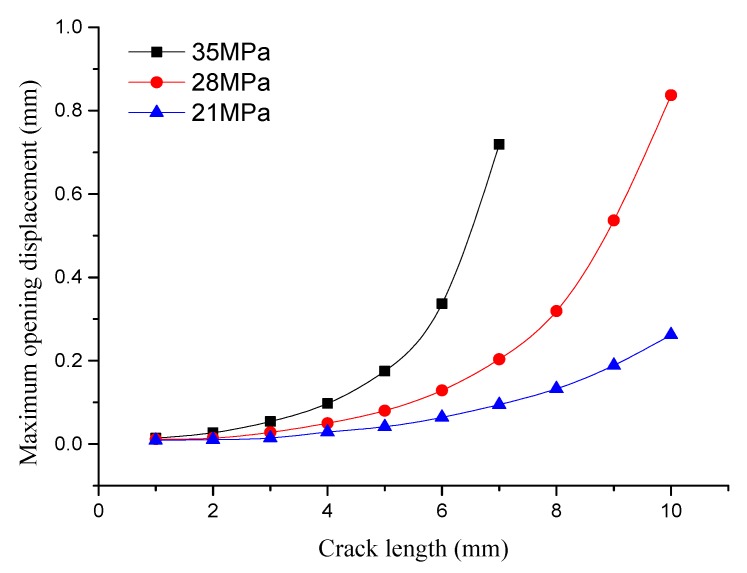
Relationship of the maximum opening displacement and the initial crack length under different internal pressures.

The displacement of the middle part of the crack also changes during the change of crack length. The relationship of the maximum crack displacement and initial crack length in the middle part of the crack is shown in Figure 11. It can be seen that the maximum opening displacement of the crack continues to increase when the crack length increases. At the same time, it can be seen that the change rate of the maximum opening displacement in the crack midpoint increases with the increase of crack length. When the initial crack length increases to 9 mm under an internal pressure of 28 MPa, the maximum opening displacement of the crack midpoint reaches 0.5369 mm. At this time, the pipe still has certain load-carrying capacity. 

When the initial crack length increases to 10 mm, the pipe crack enters the free development stage and the pipe completely loses load-carrying capacity. The maximum opening displacement of the crack midpoint reaches 0.837 mm at this time. The opening displacement of the crack reaches 0.719 mm when the initial crack length increases to 7 mm under an internal pressure of 35 MPa. At this moment, the pipe loses load-carrying capacity and the opening displacement of the crack midpoint reaches its critical value.

Here, we assume the initial crack length is $l_{0}$ and the half-crack propagation length is $l_{1}$. The crack propagation rate Δ can be expressed as

(11) $$\Delta =\frac{2l_{1}}{l_{0}}$$

The calculation results of the crack propagation rate are given in Table 4. It can be seen that the crack length starts to expand beyond the critical state when the initial crack length reaches 4 mm under an internal pressure of 21 MPa. The crack propagation rate at this time is 50.75%. As the initial crack length increases, the trend of propagation speed increases faster. When the initial crack length increases to 10 mm, the crack propagation rate is 96.46%. Under an internal pressure of 28 MPa, the initial crack length, which begins to propagate, is shorter than that at 21 MPa. However, the crack propagation rate increases more obviously, especially when the initial crack length exceeds 7 mm. The propagation rate even exceeds 200% when the initial crack length exceeds 9mm. At this time, the pipe has lost its load-carrying capacity. Under an internal pressure 35 MPa, the initial crack length, which begins to propagate, is shorter and the propagation rate is more intense than in the above two cases. When the initial crack length is 7 mm, the propagation rate of crack reaches 435.14%, which is far greater than the critical crack length of the pipe. The pipe completely loses its load-carrying capacity under an internal pressure of 35 MPa. Therefore, crack propagation rates for 8 mm, 9 mm, and 10 mm initial crack lengths are not given in Table 5.

## 4. Factors Affecting Crack Propagation

**Figure 12:**
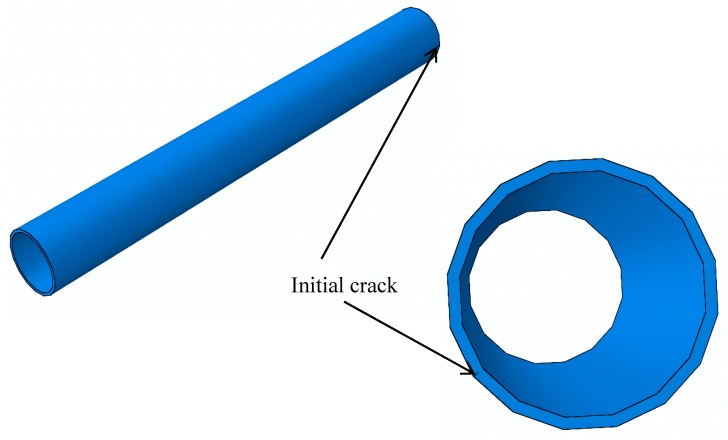
Geometrical model of a hydraulic pipe with a semi-penetrating initial crack.

**Figure 13:**
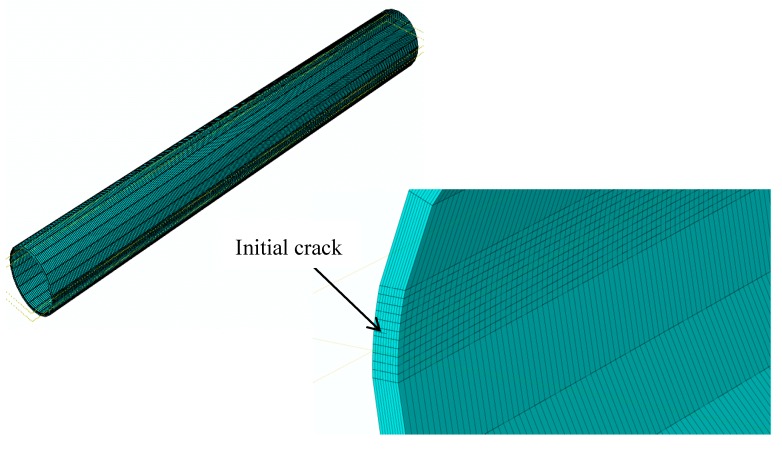
Finite element model of a hydraulic pipe with a semi-penetrating initial crack.

The geometric models involved in this section are divided into two categories: the outer wall of the hydraulic pipe contains one crack, and the inner wall of the hydraulic pipe contains another crack. Stress and strain fields of cracks without penetration are studied in this section. All of the following hydraulic pipes and initial cracks are modeled using a symmetric boundary in order to ensure that the crack depth direction can be clearly and visually displayed. All pipes have the same radius. The wall thickness corresponds to Section 3.1 (i.e., it is half of the original length). The initial crack length is also reduced to half of the original length. The geometric model and finite element model are shown in Figure 12 and Figure 13, respectively. 

Figure 13 shows a hydraulic pipe geometry model with a semi-penetrating initial crack, where radius is 8 mm, the wall thickness is 0.8 mm, and the crack depth is 0.4 mm. As symmetrical boundaries are used, the model size is only half of the actual size. In order to avoid repetition, other geometric models of crack depth and wall thickness conditions are not listed here. In this model, both ends of the pipe are fixed and the boundary in the middle part of the pipe is symmetrical. Mechanical parameters of materials used in the finite element model are given in Table 3. The difference between a semi-penetrating crack and a complete penetrating crack is that a semi-penetrating crack can be expanded in two directions, namely, longitudinal and radial directions. Therefore, crack propagation in the longitudinal direction and radial direction will be analyzed partially to explore the factors that affect crack propagation. The crack propagation priority in the longitudinal and radial directions will also be studied.

In this section, the geometric radius of the pipe is 8 mm and the wall thickness is 0.8 mm. The initial crack lengths are 1 mm, 2 mm, 3 mm, 4 mm, 5 mm, 6 mm, 7 mm, 8 mm, 9 mm, and 10 mm. The depths of the initial crack are 0.16 mm, 0.32 mm, 0.4 mm, 0.48 mm, and 0.64 mm. The working conditions are 21 MPa, 28 MPa, and 35 MPa. The initial crack location is either the outer surface or the inner surface. There are a total of 300 calculation models.

### 4.1. Factors Affecting Initial Crack Propagation on the Outer Surface 

#### 4.1.1. Crack Propagation in the Longitudinal Direction

**Figure 14:**
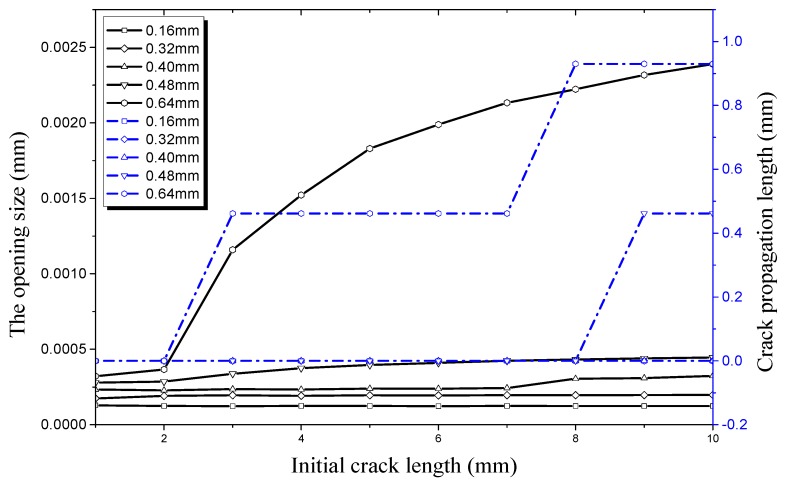
Relationship between longitudinal crack propagation length and initial crack length on the outer surface, and relationship between longitudinal opening size and initial crack length on the outer surface, both under an internal pressure of 21 MPa.

The relationship of longitudinal crack propagation length and initial crack length on the outer surface with that of longitudinal opening size and initial crack length on the outer surface is shown in Figure 14 under an internal pressure of 21 MPa. It can be obviously seen that the opening displacement shows little change no matter how the initial crack length changes when crack depth is small, especially when it is 0.16 mm or 0.32 mm. This shows that a pipe with a shallow crack is still in a stable state under an internal pressure of 21 MPa. The initial size of the crack changes slightly when the initial crack depth increases to 0.4 mm. However, it still does not reach its critical value, the crack length curve is still a straight line. When the crack depth increases to 0.48 mm, the crack propagation curve increases sharply when the initial crack length exceeds 8 mm. It can be seen that the working condition at this time reaches a critical value when the crack begins to propagate. The opening displacement of the initial crack in the longitudinal direction continues to increase when the initial crack depth is 0.64 mm. When the initial crack length increases to 2 mm it reaches a critical value, leading the initial crack into the rapid expansion period. When the initial crack length increases to 7 mm, the crack propagation enters the expansion period again. The crack propagation also ends soon after and enters the plateau, which is the same as the previous stage.

**Figure 15:**
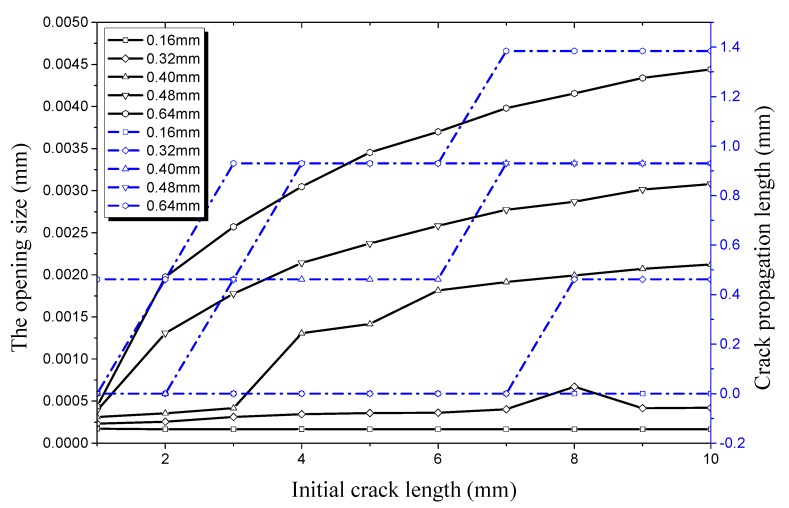
Relationship between longitudinal crack propagation length and initial crack length on the outer surface, and relationship between longitudinal opening size and initial crack length on the outer surface, both under an internal pressure of 28 MPa.

Compared Figure 15, it can be seen in Figure 15 that the crack propagation length curve remains basically unchanged no matter how the initial crack length changes when the crack depth is 0.16 mm under an internal pressure of 28 MPa. This indicates that the strength, stiffness, and load-carrying capacity of the hydraulic pipe does not show a significant decline in this condition; the pipe is still in a safe state. The initial crack begins to propagate when the crack depth is 0.32 mm, with the crack length increasing to 8 mm. This indicates that the hydraulic pipe enters a critical state under this condition. When crack depth increases to 0.40 mm, 0.48 mm, and 0.64 mm, the pipe with the initial crack experiences rapid crack propagation and a plateau period. It can be seen from Figure 15 that the critical length of the crack entering the propagation period is 8 mm and the opening size in the longitudinal direction is 6.697 × 10^−4^ mm when crack depth is 0.32 mm. The initial crack enters the germination period when the crack depth is 0.40 mm and the crack length increases to 3 mm. At this time, the opening size in the longitudinal direction is 4.16 × 10^−4^ mm. When the crack depth increases to 0.48 mm and 0.64 mm, the critical length of the initial crack entering the propagation period reduces to 2 mm and then 1 mm, respectively. This indicates that the critical length of the initial crack entering the propagation period continues to decrease when the initial crack depth increases. Then effect of initial crack depth on the load-carrying capacity of the pipe is obvious, especially when the initial crack depth is 0.64 mm. In this condition, the critical opening displacement and critical crack length are smaller, and are sensitive to external pressure. 

**Figure 16:**
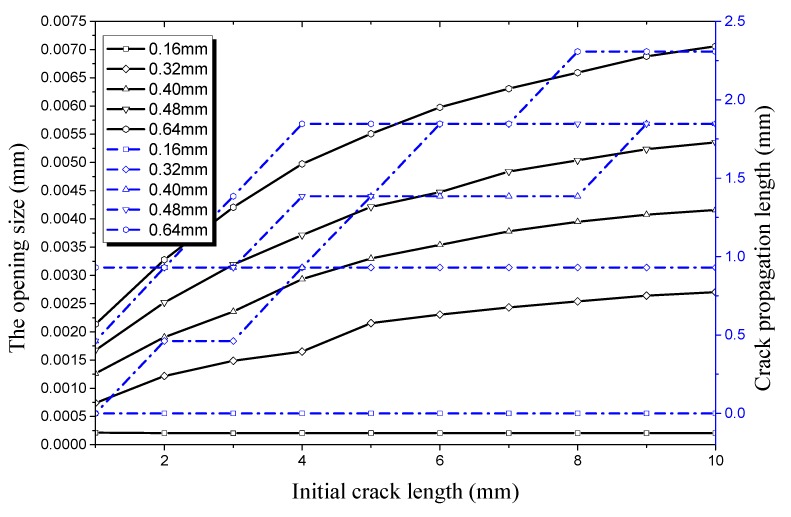
Relationship between longitudinal crack propagation length and initial crack length on the outer surface, and relationship between longitudinal opening size and initial crack length on the outer surface, both under an internal pressure of 35 MPa.

It can be seen from Figure 16 that the trend of crack propagation is the same as that when the crack depth is 0.16 mm; only the numerical values are different. When the crack depth is 0.32 mm, the critical length of the initial crack propagation is 2 mm and the crack opening displacement in the longitudinal direction is 0.4615 mm. in other conditions, the critical length of the initial crack increases to 1 mm with an increase of crack depth. This shows that the critical threshold value is further reduced under an internal pressure of 35 MPa. At the same time, the crack opening size in the longitudinal direction seems to increase obviously. However, the increase rate of the crack size slows down with increasing initial crack length, while the percentage of the crack propagation rate decreases with increasing initial crack length. It can be seen that the crack propagation rate will decline within a certain range because of the increase of free surface area when the crack length increases.

#### 4.1.2. Crack Propagation in the Radial Direction

**Figure 17:**
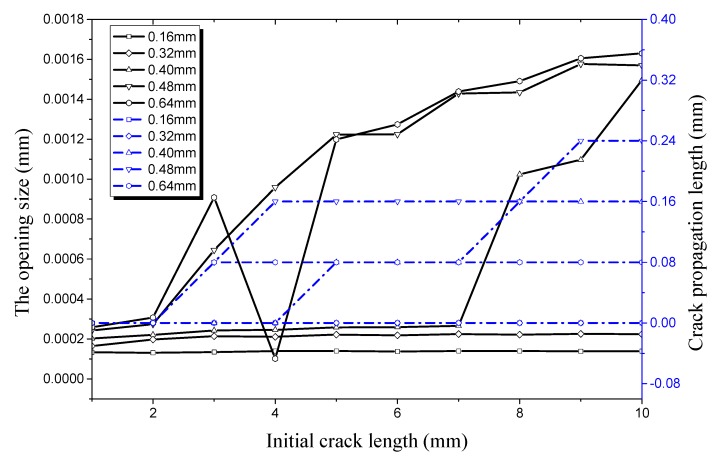
Relationship between radial crack propagation length and initial crack length on the outer surface, and relationship between radial opening size and initial crack length on the outer surface, both under an internal pressure of 21 MPa.

The relationship of radial crack propagation length and initial crack length on the outer surface with that of radial opening size and initial crack length on the outer surface is shown in Figure 17 under an internal pressure of 21 MPa. It can be seen that the radial crack propagation length first increases obviously with the increase of crack depth, and then decreases. When the crack depth is 0.16 mm and then 0.32 mm, the maximum propagation length is 2.26 × 10^−4^ mm. At the same time, the crack does not propagate along the radial direction no matter how much the initial crack length increases. When the crack depth increases to 0.40 mm, the crack begins to propagate along the radial direction as the initial crack length increases. The initial crack of the pipe enters a germination period when the initial crack length increases to 5 mm. Then, crack propagation increases to 0.16 mm in the radial direction when the initial crack length increases to 8 mm. At this time, the maximum crack growth rate is 40%. When the initial crack depth increases to 0.48 mm, the critical initial crack length at which the crack begins to propagate is 3 mm, and the maximum crack propagation length is 0.24 mm. At this time, the crack propagation rate reaches 50%, showing a significant increase compared with previous crack depths. When the initial crack depth increases to 0.64 mm, the critical length of the initial crack is still 3 mm, while the maximum crack propagation length decreases to 0.08 mm and the propagation rate decreases to 12.5%. Thus, the radial propagation rate first increases and then decreases when the crack depth increases. In the process of crack propagation, none of the cracks penetrate the wall of the hydraulic pipe. This shows that the hydraulic pipe still has a certain load-carrying capacity. Comparing Figure 15 with Figure 17, it can be seen that the length of the initial crack propagating along the radial direction is smaller than that propagating along the longitudinal direction for the same initial crack depth. 

**Figure 18:**
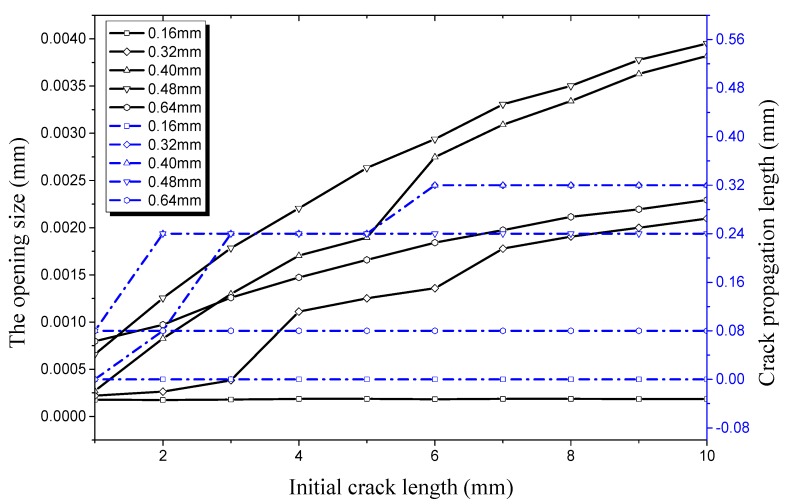
Relationship between radial crack propagation length and initial crack length on the outer surface, and relationship between radial opening size and initial crack length on the outer surface, both under an internal pressure of 28 MPa.

Compared with Figure 17, there is a significant change in Figure 18. As the internal pressure increases, the critical crack depth decreases as the crack begins to propagate along the radial direction. However, the extreme point of the crack radial propagation rate increases earlier. It can be seen from Figure 18 that no radial propagation occurs when the crack depth is 0.16 mm. When the crack depth increases to 0.32 mm and the crack length increases from 1 mm to 2 mm, the crack propagation length in the radial direction is 0.08 mm and the crack propagation rate reaches 25%. With the increase of initial crack depth and length, the maximum crack propagation rate along the radial direction reaches 94.12%, the crack depth is 0.32 mm, and the crack length is 6 mm. At this time, the opening displacement of the crack is 1.36 × 10^−3^ mm. When the crack depth is 0.40 mm, the maximum opening displacement along the radial direction is 3.82 × 10^−3^ mm, the crack propagation length is 0.32mm, and the crack propagation rate reduces to 80%. When the crack depth is 0.48 mm, the maximum radial opening displacement is 3.95 × 10^−3^ mm. The radial propagation rate of the crack drops sharply to 50% and reaches 12.5% when the crack depth is 0.64 mm. It can be seen from Figure 15 and Figure 18 that the difference in the cracking points along both radial and longitudinal directions is further reduced. With the increase of internal pressure, the critical cracking conditions approach and the difference of crack propagation decreases in both radial and longitudinal directions. It can be seen that cracking in both the radial and longitudinal directions increase rapidly. 

**Figure 19:**
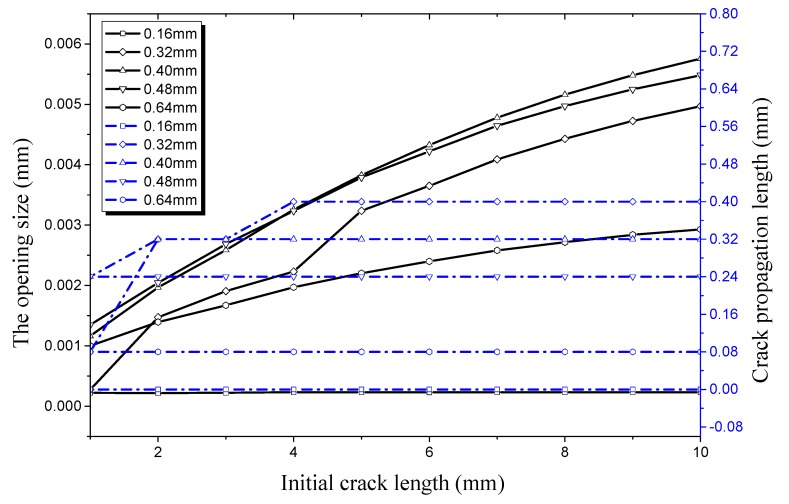
Relationship between radial crack propagation length and initial crack length on the outer surface, and relationship between radial opening size and initial crack length on the outer surface, both under an internal pressure of 35 MPa.

The effect of initial crack depth and length on radial crack propagation is shown in Figure 19 under internal pressure 35 MPa, along with the relationship between crack propagation length and radial opening size. It can be seen from Figure 16 and Figure 19 that the distance between the points where the initial crack begins to propagate along the radial direction and that along the longitudinal direction is further reduced. In this condition, the critical point of radial crack propagation is located at 0.32 mm in depth and 1 mm in length. The critical point of crack propagation along the longitudinal direction is located at approximately 0.32 mm in depth and 2 mm in length. At the same time, the maximum crack propagation rate is 125% and the critical radial opening displacement is 2.23 × 10^−3^ mm when the crack depth is 0.32 mm. The overall critical opening displacement is 2.75 × 10^−4^ mm and the length is 1 mm when the crack depth is 0.32 mm. Numerical simulation results show that the maximum depth of crack propagation is 0.72 mm when the internal pressure is 35 MPa. At this time, the pipe still has load-carrying capacity, even under the maximum internal pressure.

### 4.2. Factors Affecting Initial Crack Propagation on the Inner Surface 

#### 4.2.1. Crack Propagation along the Longitudinal Direction

**Figure 20:**
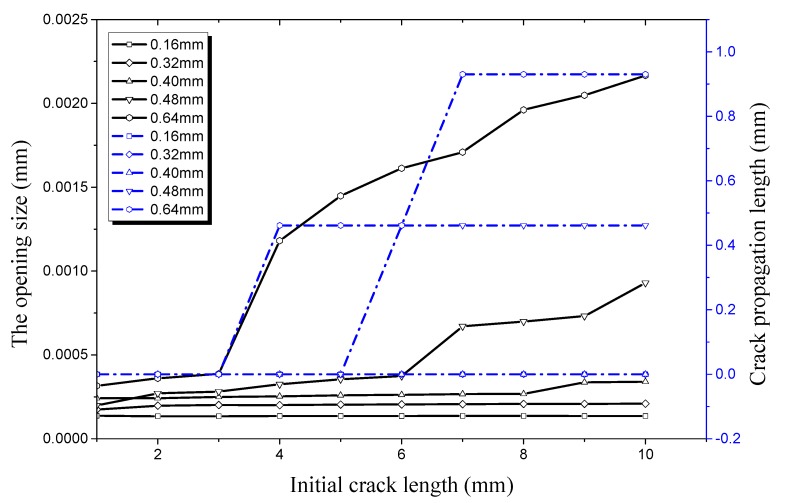
Relationship between longitudinal crack propagation length and initial crack length on the inner surface, along relationship between longitudinal opening size and initial crack length on the inner surface, both under an internal pressure of 21 MPa.

As shown in Figure 20, crack propagation occurs when the opening size increases from 0 to 3.74 × 10^−4^ mm along the longitudinal direction. At this time, the initial crack depth is 0.48 mm and the length is 6 mm. The critical opening size along the longitudinal direction is 1.18 × 10^−3^ mm when the crack depth is 0.64 mm and the length is 4 mm. This shows that it becomes more difficult to for the crack propagate along the longitudinal direction with the increase of crack depth, local freedom degree of the hydraulic pipe, and the critical value of crack propagation. By comparing the critical points of crack propagation (shown in Figure 14 and Figure 20), it can be seen that the critical crack length on the inner surface is 6 mm and that on the outer surface is 9 mm when crack depth is 0.48 mm. When the crack depth is 0.64 mm, the critical crack length on the inner surface is 4 mm and that on the outer surface is 3 mm. This shows that the propagation of the inner crack is more rapid than that of the outer crack, and that the damage is more obvious when the crack depth is relatively small. However, the outer crack propagates more rapidly than the inner crack as the crack depth continues to increase. It can be seen from Figure 20 that the critical opening size along the longitudinal direction is 3.74–4 mm when the crack depth is 0.48 mm, and it is 1.18 × 10^−3^ mm when the crack depth is 0.64 mm. Comparing both of these cases, it can be seen that the critical opening size increases by 2.18 times while the crack depth increases by only 33.3%. This is caused by the fact that freedom degrees of the crack surface increase with crack depth. Otherwise, attenuation of the actual bearing pressure leads to a sharp increase of the deformation, and thereby greatly increases the critical opening size. 

**Figure 21:**
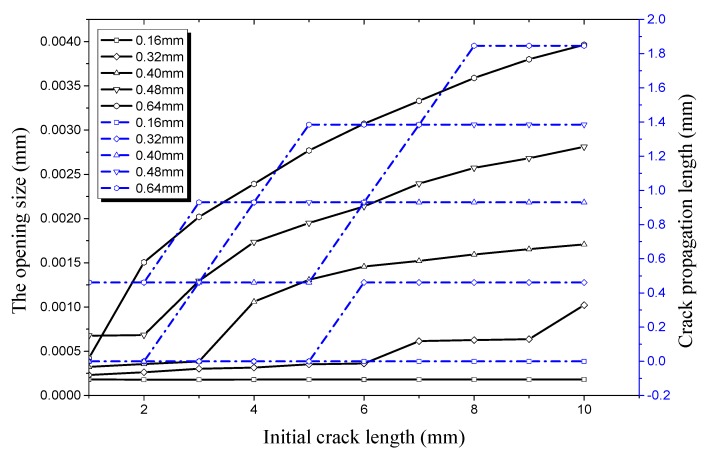
Relationship between longitudinal crack propagation length and initial crack length on the inner surface, and relationship between longitudinal opening size and initial crack length on the inner surface, both under an internal pressure of 28 MPa.

When the crack depth is 0.16 mm, it can be seen from Figure 21 that the opening displacement along the longitudinal direction remains substantially unchanged as the crack length increases from 1 mm to 10 mm. This causes the crack propagation length to be unchanged. It can be deduced that the opening displacement along the longitudinal direction has not yet reached the critical value for the initial crack at this time. When the crack depth increases to 0.32 mm, the longitudinal displacement curve begins to change significantly. The opening size along the longitudinal direction increases to 3.62 × 10^−4^ mm when the crack length increases to 6 mm. At this time, the initial crack begins to enter the propagation stage and the crack propagation value is 0.4615 mm. The displacement change curve is more obvious when the crack depth increases to 0.40 mm. The critical crack length at which the crack begins to propagate decreases from 6 mm at 0.32 mm initial crack depth to 3 mm at 0.40 mm initial crack depth, while the critical value of the longitudinal displacement increases to 3.85 × 10^−4^ mm. When the crack depth increases to 0.48 mm, the critical value of the opening displacement along the longitudinal direction becomes 6.83 × 10^−4^ mm and the initial crack length is 2 mm at this time. When the crack depth increases to 0.64 mm and the initial crack length is 2 mm, the opening size along the longitudinal direction is 1.51 × 10^−3^ mm. It can be seen that there is a significant increase of critical opening displacement along the longitudinal direction with the increase of the crack depth under an internal pressure of 28 MPa. 

**Figure 22:**
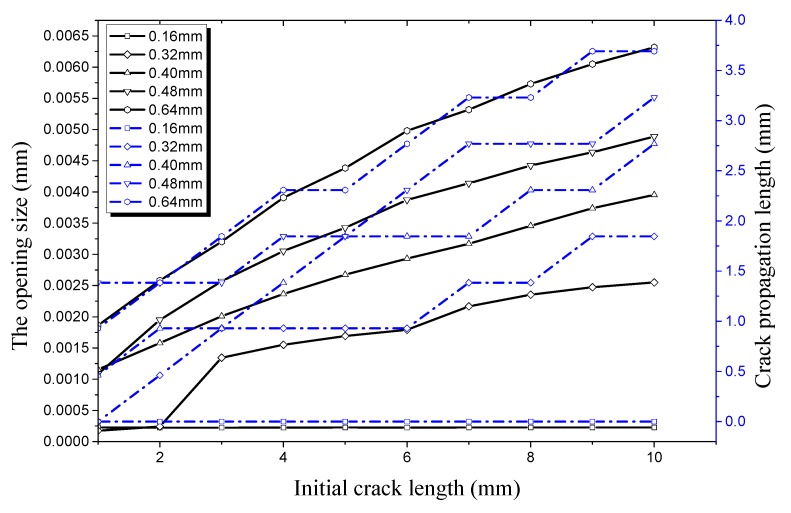
Relationship between longitudinal crack propagation length and initial crack length on the inner surface, and relationship between longitudinal opening size and initial crack length on the inner surface, both under an internal pressure of 35 MPa.

When the crack depth is 0.16 mm, it can be seen from Figure 22 that the opening displacement along the longitudinal direction does not reach the critical value, since the crack propagation length is 0 mm. This shows that the pipe is in the safe stage when the initial crack depth is 0.16 mm. When the initial crack depth is 0.32 mm, the crack begins to propagate as the longitudinal opening displacement reaches 2.42 × 10^−4^ mm with a propagation length of 0.4615 mm. The initial crack length is 2 mm at this time. When initial crack depth is 0.40 mm, the critical opening size in the longitudinal direction reaches 1.16 × 10^−^^3^ mm and there is a clear jump in longitudinal opening displacement. When the initial crack depth is 0.64 mm, the critical opening size in longitudinal direction increases to 1.86 × 10^−^^3^ mm, the critical propagation length is 0.9302 mm, and propagation rate reaches 93.02%. Simulation results under an internal pressure of 35 MPa show that crack propagation length is inversely correlated with crack depth, while the critical opening displacement of the crack is positively correlated with crack depth.

#### 4.2.2. Crack Propagation along the Radial Direction

**Figure 23:**
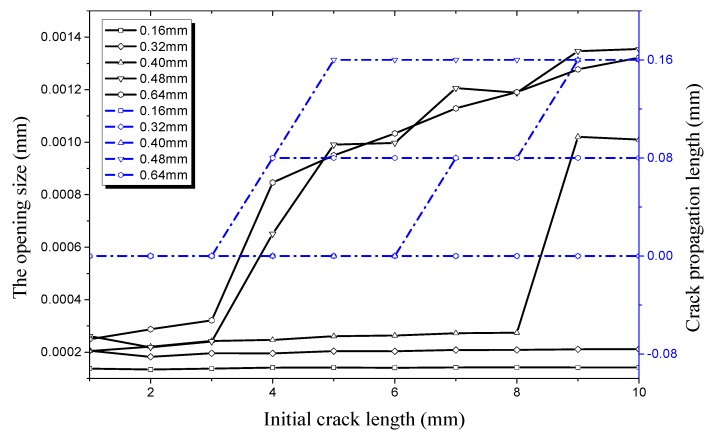
Relationship between radial crack propagation length and initial crack length on the inner surface, and relationship between radial opening size and initial crack length on the inner surface, both under an internal pressure of 21 MPa.

The effect of initial crack size on crack opening displacement and propagation length is shown in Figure 23 under an internal pressure of 21 MPa. It can be seen that the radial opening displacement increases slowly and the crack propagation length remains at 0 mm at crack depths of 0.16 mm and 0.32 mm. Thus, the crack does not propagate along the radial direction when the crack depth is 0.16 mm or 0.32 mm. The crack begins to propagate along the radial direction when the crack depth increases to 0.40 mm and the crack length increases to 7 mm. At this time, the radial opening displacement of the crack is 2.72 × 10^−^^4^ mm and the crack radial propagation rate is 20%. The initial crack begins to propagate radially when the crack depth increases to 0.48 mm with a crack length of 4 mm. The radial displacement of the crack is 6.51 × 10^−^^4^ mm and the crack radial propagation rate is 16.7%. When the crack depth increases to 0.64 mm with a crack length of 4 mm, the crack begins to propagate radially. At this time, the radial opening displacement of the crack is 8.46 e/4 mm and the crack radial propagation rate is 12.5%. From the above analysis, it can be seen that the critical opening displacement of the crack also appears to increase with the increase of the crack depth. This is caused by the fact that the actual pipe thickness bearing the inner pressure becomes thinner and the deformation is relatively increased under internal pressure when the crack depth increases. The radial propagation rate of the crack decreases at the same time. Comparing Figure 23 with Figure 20, the critical point of crack propagation along the radial direction occurs earlier than that along the longitudinal direction. The propagation length of the crack increases rapidly when the crack propagates along the radial direction. This reduces the stress concentration in the radial direction and leads to the decrease of the radial crack propagation rate.

**Figure 24:**
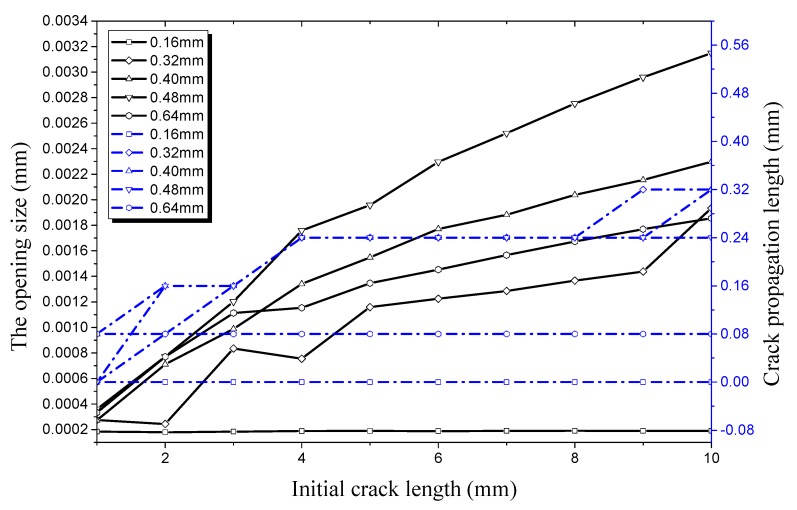
Relationship between radial crack propagation length and initial crack length on the inner surface, and relationship between radial opening size and initial crack length on the inner surface, both under an internal pressure of 28 MPa.

It can be seen from Figure 24 that the crack begins to propagate when the crack depth is 0.32 mm, which is different from the radial propagation under an internal pressure of 21 MPa. The crack propagates radially when the initial crack length reaches 2 mm with a radial opening displacement of 2.44 × 10^−^^4^ mm. When the initial crack depth increases to 0.40 mm with an initial crack length of 1 mm, radial propagation occurs; the opening displacement along the radial direction is 2.72 × 10^−^^4^ mm. When the initial crack depth increases to 0.48 mm and then to 0.64 mm, the radial opening displacement of crack increases further to a maximum value of 3.33 × 10^−^^4^ mm. The radial crack propagation rate decreases sharply from the maximum value of 80% to less than 15%. By comparing Figure 24 with Figure 21, the critical point of the radial crack propagation occurs earlier than that of the longitudinal crack propagation. The crack radial propagation rate increases with the increase of initial crack length, while it decreases with the increase of initial crack depth. Crack propagation along the longitudinal direction greatly affects the crack radial propagation. Comparing Figure 18 with Figure 24, it can be seen that the propagation rates of outer surface cracks are generally greater than that of inner surface cracks under the same conditions, except in the case where the initial crack depth is 0.64 mm. This indicates that the destructiveness of outer surface cracks exceeds that of inner surface cracks. 

**Figure 25:**
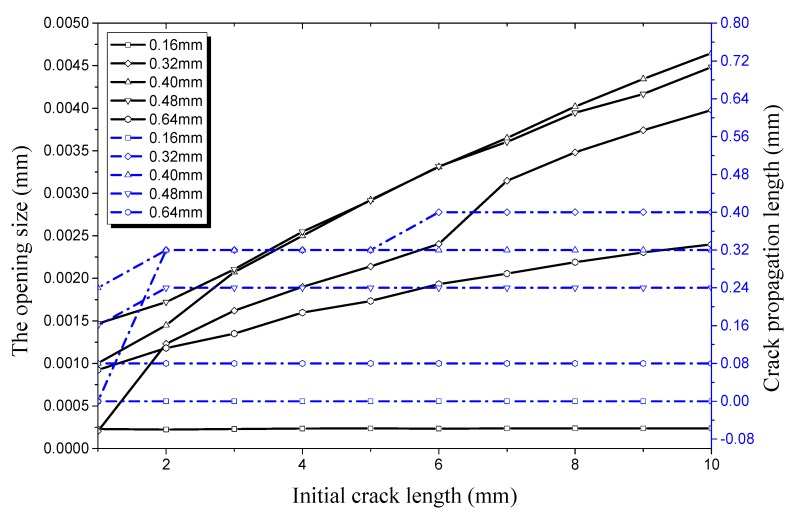
Relationship between radial crack propagation length and initial crack length on the inner surface, and relationship between radial opening size and initial crack length on the inner surface, both under an internal pressure of 35 MPa.

It can be seen from Figure 25 that the crack propagation length is 0 mm when the initial crack depth is 0.16 mm. No crack propagation occurs in the longitudinal direction when comparing with Figure 22. This indicates that the crack is stable in both radial and longitudinal directions when the crack depth is 0.16 mm. Numerical simulation results under an internal pressure of 35 MPa are shown in Figure 22 and Figure 25. It can be seen that no crack propagation occurs in the pipe when the crack depth is 0.16 mm, and its working condition is still in the safe stage. When the crack length increases to 0.32 mm, the opening displacement of the crack increases sharply in the radial direction, and its critical value reaches 1.23 × 10^−^^3^ mm. The propagation rate reaches 100% in the radial direction. The radial opening displacement decreases when the crack depth increases from 0.40 mm to 0.64 mm. This indicates that the crack propagation rate exceeds the deformation rate of the pipe under high internal pressure. Compared with Figure 22, the critical value of crack propagation along the radial direction occurs earlier than that in the longitudinal direction. The crack propagation rate in the radial direction enters the decline stage, affected by crack propagation in the longitudinal direction.

## 5. Conclusions

In this paper, the extended finite element method (XFEM) is applied to simulate the initial crack propagation in a hydraulic pipe and investigate its influence factors. The following main conclusions can be drawn.

(1) When crack depth is no more than 40% of the wall thickness, the change of initial crack length has little effect on crack propagation in a hydraulic pipe with a semi-penetrating crack. When crack depth exceeds 40% of the wall thickness, the crack propagates in radial and longitudinal directions when the initial crack length and depth change. When the crack depth exceeds 50% of the wall thickness, the crack enters a rapid expansion period. Thus, initial crack depth should be given more attention in malfunction mechanism analyses of aircraft hydraulic pipes.

(2) In the longitudinal direction of crack propagation, the crack propagation rate increases with the increase of initial crack length. In the radial direction of crack propagation, the crack propagation rate first increases and then decreases with the increase of initial crack depth. By comparing the crack propagation along the longitudinal and radial directions under the same conditions, it can be seen that the crack expands to a certain extent first in the radial direction and then expands in the longitudinal direction. This implies that radial propagation is more destructive than longitudinal propagation in hydraulic pipes in the initial stage of crack propagation. Therefore, radial propagation of the initial crack should be further researched in hydraulic pipe structures that contain initial cracks.

The XFEM can be accurately used to simulate gradient changes of crack stress–strain fields and can clearly show the stress transmission path and stress concentration in the crack tip. This conclusion is consistent with other research by XFEM [32], and the verified method provides a robust and versatile numerical tool to solve crack problems in hydraulic pipe structures. However, there are some drawbacks of the current work. The problem that crack propagation length must be an integral multiple of the unit length has not been solved in the current work. In addition, arbitrary length crack propagation and dynamic crack propagation should be researched in the future.

## Figures and Tables

**Table 1:** Material properties of the rectangular plate.

E (GPa)	μ	Maximum Principal Stress (MPa)	G_I_ (N/m)	G_II_ (N/m)	G_III_ (N/m)
210	0.3	84.4	42,200	42,200	42,200

**Table 2:** Stress intensity factors F for different plate sizes.

**Ratio of Initial Crack a/b**	0.0625	0.125	0.1875	0.25	0.3125	0.375	0.4375	0.5
**F**	1.142	1.222	1.344	1.505	1.711	1.981	2.345	2.843

**Table 3:** The von Mises stress distribution of the rectangular plate for different crack lengths and mesh sizes.

	Crack Length	0.5 m	2 m	4 m
Mesh Size				
0.3 m		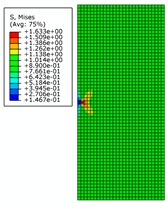	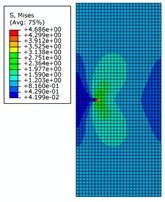	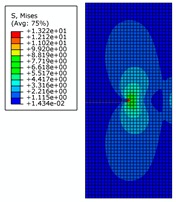
0.4 m		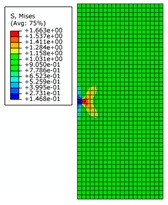	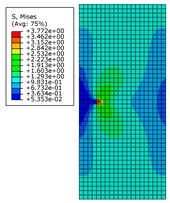	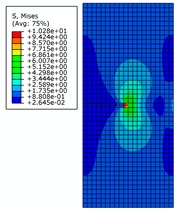
0.8 m		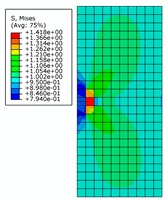	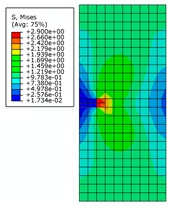	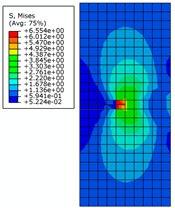

**Table 4:** Material properties of the hydraulic pipe.

E (GPa)	μ	Maximum Principal Stress (MPa)	G_I_ (N/m)	G_II_ (N/m)	G_III_ (N/m)
210	0.3	540	643,000	643,000	643,000

**Table 5:** Crack propagation rates Δ and half-crack propagation length *l*_1_ for different initial crack length *l*_0_ and internal pressures.

	*l*_0_/mm	1 mm	2 mm	3 mm	4 mm	5 mm	6 mm	7 mm	8 mm	9 mm	10 mm
Internal Pressure/MPa											
21	*l*_1_/mm	0	0	0	1.015	1.015	1.523	2.03	3.045	3.553	4.823
	Δ/mm	0	0	0	50.75	40.6	50.77	58	76.125	78.96	96.46
28	*l*_1_/mm	0	0	1.015	2.03	2.5375	3.5525	4.5675	7.105	10.6575	13.195
	Δ/mm	0	0	67.67	101.5	101.5	118.42	130.5	177.625	236.83	263.9
35	*l*_1_/mm	0	1.523	3.046	4.1615	5.5825	9.138	15.23	—	—	—
	Δ/mm	0	152.3	203.07	208.075	223.3	304.6	435.14	—	—	—
